# Plutonium from Above-Ground Nuclear Tests in Milk Teeth: Investigation of Placental Transfer in Children Born between 1951 and 1995 in Switzerland

**DOI:** 10.1289/ehp.11358

**Published:** 2008-09-17

**Authors:** Pascal Froidevaux, Max Haldimann

**Affiliations:** 1 University Institute of Radiation Physics, University Hospital Center, University of Lausanne, Lausanne, Switzerland; 2 Consumer Protection, Chemical Risks, Federal Office of Public Health, Bern, Switzerland

**Keywords:** fetus exposure, milk teeth, nuclear bomb test fallout, placenta transfer, plutonium, plutonium metabolism

## Abstract

**Background:**

Occupational risks, the present nuclear threat, and the potential danger associated with nuclear power have raised concerns regarding the metabolism of plutonium in pregnant women.

**Objective:**

We measured plutonium levels in the milk teeth of children born between 1951 and 1995 to assess the potential risk that plutonium incorporated by pregnant women might pose to the radiosensitive tissues of the fetus through placenta transfer.

**Methods:**

We used milk teeth, whose enamel is formed during pregnancy, to investigate the transfer of plutonium from the mother’s blood plasma to the fetus. We measured plutonium using sensitive sector field inductively coupled plasma mass spectrometry techniques. We compared our results with those of a previous study on strontium-90 (^90^Sr) released into the atmosphere after nuclear bomb tests.

**Results:**

Results show that plutonium activity peaks in the milk teeth of children born about 10 years before the highest recorded levels of plutonium fallout. By contrast, ^90^Sr, which is known to cross the placenta barrier, manifests differently in milk teeth, in accordance with ^90^Sr fallout deposition as a function of time.

**Conclusions:**

These findings demonstrate that plutonium found in milk teeth is caused by fallout that was inhaled around the time the milk teeth were shed and not from any accumulation during pregnancy through placenta transfer. Thus, plutonium may not represent a radiologic risk for the radiosensitive tissues of the fetus.

Most of the plutonium (Pu) present in the environment can be traced back to fallout from nuclear bomb testing. The legacy from above-ground testing involves not only Pu but also other radionuclides such as cesium-137 (^137^Cs), strontium-90 (^90^Sr), tritium (^3^H), and carbon-14 (^14^C). Air concentrations of all these pollutants show a dramatic rise between 1950 and 1963, with a large amount of transuranic elements and fission products injected into the atmosphere during 1960–1962. Subsequent years were marked by an exponential decrease of these elements, caused by the adoption of the Nuclear Test Ban Treaty in 1963, absorption into the biosphere, and radioactive decay [Froidevaux et al. 2006; Kelley et al. 1999; Spalding et al. 2005; U.N. Scientific Committee on the Effects of Atomic Radiation (UNSCEAR) 2000; Warneke et al. 2002]. Several studies indicate that some of this Pu eventually found its way into human beings [Franke et al. 1995; Ibrahim et al. 2002; International Commission on Radiological Protection (ICRP) 1986; Kathren 2004; O’Donnell et al. 1997; Taylor 1995]. There are also a few cases of accidental contamination of humans with Pu via wounds or the inhalation of particles in the workplace (Daniels et al. 2006; Filipy et al. 1994; Kathren 2004; Krahenbuhl et al. 2005; Russell et al. 2003). The current systemic biokinetic model for Pu in humans recommended by the ICRP (1993) has been discussed in Leggett (2003), commenting that extrapolating data obtained from laboratory animals onto humans might not be reliable, particularly for the liver, because of differences among species. The general observation is that Pu entering the systemic circulation distributes in equal parts to the liver and the skeleton. In this context, Pu can be considered a bone-seeking radionuclide.

Present dangers associated with Pu apart from occupational risks include nuclear weapon proliferation, an increasing risk of nuclear terrorism, and the 35,000 warheads that remain in the nuclear arsenals of the world’s superpowers (Forrow and Sidel 1998). The civil use of nuclear energy will contribute to Pu dissemination at short distances, from accidents such as the one that occurred in Chernobyl or from leakage in waste repositories. An example of involuntary mishandling of Pu-containing material is represented by trinitite, a mineral formed during the blast of the first atomic bomb in 1945 that fused the desert sand around ground zero in Alamogordo, New Mexico. This material is sold freely on the Internet and contains as much as 100,000 Bq Pu/kg (Parek et al. 2006). Thus, just 0.5 g trinitite exceeds most national regulations on radioprotection and should necessitate special authorization to handle it.

Pregnant women are exposed to a variety of toxic substances coming from either environmental or occupational exposure. The placenta provides the link between a mother and a fetus, although its main task is to transport nutrients and oxygen to the fetus. In addition, it acts as a barrier against foreign compounds. However, some toxic substances may be transported across the placenta to some degree and may therefore have an impact on the unborn child. Establishing whether Pu can cross the placental barrier is an important task in view of a fetus’s particularly high radio-sensitivity. Indeed, Pu has often been regarded as a highly radiotoxic element spread worldwide via human activities (Bair and Thomson 1974; Franke et al. 1995; Ibrahim et al. 2002; ICRP 1986; Kathren 2004; O’Donnell et al. 1997; Taylor 1995). The genetic risk from exposure to ionizing radiation is attributed mostly to DNA deletion, a phenomenon that often encompasses multiple genes (Sankaranarayanan and Wassom 2003). After intrauterine irradiation, it is thought that even a small dose (on the order of 10 mSv) can have adverse effects on the risk of childhood cancer (Wakeford and Little 2003). However, currently available data on placental transfer were obtained mostly through animal experimentation, and evidence that the placenta might behave as a barrier against Pu has been predicted but never confirmed (Mason et al. 1992; Paquet et al. 1998; Prosser et al. 1994; Russell et al. 2003; Sikov and Kelman 1989). Here we use milk teeth, whose enamel is formed during pregnancy, to examine Pu transfer from the mother’s blood plasma to the fetus. The enamel of milk teeth is deposited *in utero* and incorporates any fetal contamination received during pregnancy. For example, the ^90^Sr content of milk tooth enamel reflects the ^90^Sr levels in the environment and the food chain during pregnancy (Froidevaux et al. 2006). Sr is chemically similar to calcium and therefore crosses the placental barrier (Belkacemi et al. 2005). In a similar manner, the ^14^C content of the crown of permanent teeth has been shown to reflect the environmental ^14^C content at the time of enamel formation (Spalding et al. 2005). Gomes et al. (2004) have demonstrated that a biopsy of deciduous tooth enamel can be used to probe environmental lead contamination because lead is a bone-seeking metal cation. Likewise, Gulson and Gillings (1997) showed that the enamel of milk teeth indicates *in utero* exposure, whereas the dentine of milk teeth reveals early childhood exposure to lead. Webb et al. (2005) used human teeth and deciduous teeth to investigate dietary and environmental pollution history, in particular by measuring lead, zinc, Sr, magnesium, and Ca in tooth enamel. Tooth enamel calcifies during early development (4 months *in utero*) making teeth an excellent hard tissue for environmental and nutritional studies. Here we report a 50-year time period of Pu-239 (^239^Pu) data in the whole milk teeth of children who were born and grew up in Switzerland, at least until the age that they then shed their milk teeth.

## Materials and Methods

### Sampling

We collected deciduous teeth and vertebrae through the sampling program of the Swiss Federal Office of Public Health for environmental radioactivity survey that was initiated in conjunction with the onset of the nuclear era. We performed the first measurements (for ^90^Sr) on teeth of children born in 1950. The overall study (milk tooth and vertebra collection) was approved by the Swiss Federal Office of Public Health. Because of financial cuts, teeth were not collected for the birth years 1972 to 1980. The entire program was restarted after the Chernobyl nuclear accident (1986) and is still ongoing at the present time for ^90^Sr determination. This program has provided the Swiss population with a strong reassurance that food has not been significantly contaminated by ^90^Sr after the Chernobyl accident. The project is well received by the population. In two cases (Table 1), we roughly separated tooth crowns from the roots and measured them as independent samples, but we made no attempt to separate cementum, dentine, and enamel because of the need to obtain at least 20 g of each material to reach the detection limit of the measurement.

### Radiochemistry

Whole milk teeth were collected by dentists over the last 50 years all over Switzerland. We grouped teeth by year of birth to give at least 30 g of tooth ash (about 50 whole teeth, ashed at 550°C during 24 hr). We spiked tooth ash with 10 ± 0.5 mBq of ^242^Pu and submitted it to microwave digestion in a Milestone MLS Ethos Plus digester (MLS GmbH, Leutkirch, Germany) for 40 min at 170°C in 100 mL 8 M nitric acid. After filtration, we extracted Pu on a Bio-Rad (Reinach, Switzerland) ionic exchanger (100–200 mesh, 25 mL in a 1-cm-diameter chromatography column) and purified it on a microcolumn (100 mg) of Eichrom TEVA resin (Eichrom Environment, Bruz, France), using Ultrapur reagents (Merck, VWR International, Dietikon, Switzerland) to minimize the uranium content (we extracted Pu in 5 mL 8 M HNO_3_ and washed the column with 10 mL 3 M HNO_3_, 3 mL 9 M HCl, and once again with 3 mL 3 M HNO_3_). We measured Pu in 5% HNO_3_ by sector field inductively coupled plasma mass spectrometry (SF-ICP-MS). We used the same analytical procedure for the vertebrae. The vertebrae were collected between 1962 and 2000 by pathologists in different locations across Switzerland. We grouped them (30 g of ash) for different adult individuals who died the same year. Most (80%) of the individuals were > 60 years of age, and none were < 25 years of age at the time of death. We measured Ca on all samples by atomic absorption with a PerkinElmer 4100 apparatus (PerkinElmer AG, Schwerzenbach, Switzerland) and give the results of Pu isotope activity as Bq/g Ca.

### Mass spectrometry

We used a double focusing SF-ICP-MS (Element2; Thermo Electron, Bremen, Germany) for measuring the ^239^Pu, ^240^Pu, and ^242^Pu isotopes. We performed 36 consecutive scans on each sample. We applied the low-resolution mode (m/Δm = 300) to obtain maximum ion transmission. We equipped the instrument with an APEX Q desolvation device (Elemental Scientific, Omaha, NE, USA) in combination with an ACM membrane unit (Elemental Scientific) that was effective in removing the solvent from liquid samples, thereby retaining Pu for transport to the plasma ion source in the form of a dry aerosol. This special configuration improved the determination of Pu in two ways. First, the method improved the signal-to-noise ratios by a factor of about 10, which was necessary to reach the low Pu concentrations in teeth. Second, the method reduced the formation of the ^238^U^1^H^+^ molecular ion that interferes with ^239^Pu to a great extent. The ^238^U^1^H^+:238^U ratio was constant under the selected conditions. Therefore, we also monitored the ^238^U isotope to account for residual background contributions to the ^239^Pu signal as a result of variable uranium impurities. Optimum argon flow conditions in the PFA-100-1036 nebulizer (Elemental Scientific) were in the range of 0.9–1.1 l/min at a sample uptake rate of 235 μL/min. We ran all other aspects of the ICP-MS under normal operating conditions. We carried out quality control of chemical separation and ICP-MS measurements by spiking 100 mL of 8 M HNO_3_ with a reference solution of ^239^Pu and subjected it to the overall ^239^Pu determination process. The measured value of 2.7 ± 0.1 mBq (*n* = 7) agreed well with the reference value of 2.8 ± 0.3 mBq.

We estimated the propagation of the total measurement uncertainty based on a probabilistic model. In estimating the overall uncertainty associated with final results (activity per bone Ca mass), we quantified the uncertainties from each identified source of uncertainty—that is, all measured Pu isotopes, interfering molecular ion, Pu tracer, and Ca concentrations—as distribution functions, and then we simulated the final distributions using a Monte Carlo sampling technique (@Risk 4.5; Palisade, Newfield, NY, USA). In addition to concentration data used, we approximated high count rates (> 10 counts/sec) by a normal distribution; otherwise, we used Poisson statistics to describe the data. We obtained the standard uncertainties as parameters from the resulting distributions.

## Results

**Figure 1:**
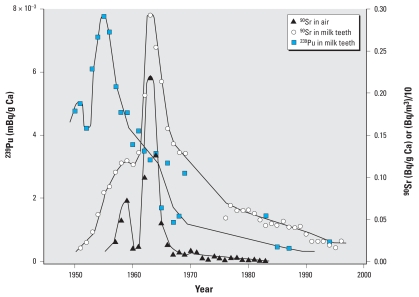
Activities of ^90^Sr and ^239^Pu in milk teeth as a function of the year of birth. Nuclear bomb tests conducted between 1955 and 1963 produced large amounts of ^239^Pu and ^90^Sr in air. ^239^Pu activities in the air can be deduced from ^90^Sr in the air [data from UNSCEAR (2000) for the Northern Hemisphere] with a factor of 1/95.

Because of the very low amount of Pu in the milk teeth, we pooled for each sample approximately 50 teeth from children born in the same year and used SF-ICP-MS techniques (Boulyga et al. 2003; Rodushkin et al. 1999) to determine the ^239^Pu at the femtogram (10^−15^ g) level. The average ^240^Pu:^239^Pu isotopic ratio was 0.21 ± 0.06 (*n* = 23), from which we inferred that the Pu source is indeed atomic bomb test fallout (Kelley et al. 1999; Warneke et al. 2002). We found that ^239^Pu activities in whole milk teeth (roots and crown) followed the time evolution of atmospheric contamination, with an offset (lag time) of about 10 years (Figure 1, Table 1). Instead of showing a maximum activity for children born in 1963, ^239^Pu activity peaks for milk teeth of children born about 10 years earlier, when the Pu activity in the environment was still low. Thus, unlike for ^90^Sr, the ^239^Pu activities in milk teeth do not reflect the environmental activities during pregnancy. We then roughly separated the roots of milk teeth from the rest of the teeth for two samples. The corresponding SF-ICP-MS determination shows that ^239^Pu in the roots has activities up to 8.5 times higher than those found in the rest of the teeth, including the enamel (Table 1). It is likely that the low “residual” Pu concentrations found in enamel samples represent contamination by residual root and dentine structures in the enamel sample. Finally, we determined the ^239^Pu activity in trabecular bones on 49 samples. These samples consisted of vertebrae of individuals who died between 1962 and 2000 in Switzerland, and we pooled them by groups of the same year of death. The isotopic ratio ^240^Pu:^239^Pu of 0.18 ± 0.01 (*n* = 49) confirms again that nuclear bomb test fallout was the origin of the Pu. When normalizing the activity of ^239^Pu to the Ca content of the sample, the peak activity in bulk milk teeth was at least 10 times lower than the peak activity in vertebrae.

## Discussion

The whole deciduous teeth as measured in this work include the enamel, dentine, pulp, and cementum. Dentine is the largest component of the root of teeth, with a lower mineral content than enamel. The pulp is the most biologically active tissue, which facilitates communication between dental tissue and the rest of the body, including nutrients. Cementum is a thin layer of calcified tissue, much like bone in its relative calcification and turnover properties, which covers the root of the tooth (Avery 1987; Webb et al. 2005).

Our interpretation of the observed time offset of Pu in milk teeth compared with Pu in the environment is that we measured the Pu within the active root zone (cementum and pulp) of the teeth, a bonelike structure, which continuously exchanges Pu with the blood plasma. We think that this part of the tooth reflects Pu air concentration during the last year or so before tooth shedding, which takes place on average 10 years after birth (10.4 ± 2 years in this study; *n* = 580). This finding is corroborated by a study about the turnover rate of Pb isotopes in deciduous teeth and the socket bone of teeth (trabecular) by Gulson and Gillings (1997), who concluded that enamel exhibits no exchange of Pb, whereas dentine Pb exchanges with its environment. The higher exchange rate was found for roots analyzed with the entire circumpulpal canal present. Finally, the authors demonstrated that Pb in trabecular bone sockets supporting teeth has been almost totally exchanged with environmental Pb isotopes over a period of 15 years. In conclusion, the authors postulate that Pb in enamel cannot migrate any farther, whereas Pb in dentine exchanges at a slow rate of about 1–2% per year and Pb in the pulp and cementum exchanges at about the same rate as trabecular socket bone, about 8% per year. Thus, our interpretation of Pu activity in deciduous teeth as a function of the year of birth is that neonates are born free of Pu and then start to incorporate Pu through inhalation after birth and until they shed their milk teeth. It would appear that, as with Pb, the circumpulpal canal and cementum will exert the most significant control on Pu concentration, leading to a higher concentration in the milk teeth shed when the Pu concentration in air was at its highest.

Therefore, it seems very probable that the enamel laid down *in utero* is virtually free of ^239^Pu. It follows that fallout Pu has not been significantly transferred from the mother to the fetus and hence to the developing milk tooth enamel. Consequently, neonates were probably born free of Pu even when the environmental Pu contamination was at its highest, in the early 1960s. These findings corroborate observations from different studies that found evidence of placental transfer discrimination against Pu (Kathren 2004; Paquet et al. 1998; Russell at al. 2003).

The conclusion that Pu in milk teeth sits in the bonelike root of the teeth and the pulp is supported by comparison with bones. O’Donnell et al. (1997) found that permanent teeth have the same activity level as bones. In this respect, both O’Donnell et al.’s work and our study demonstrate that milk teeth can be used to investigate Pu transfer from mother to fetus, because teeth are as much a target for Pu as are bones. Moreover, the comparison between both studies corroborates the finding that the main part of the apatite of milk teeth (enamel of milk teeth that was laid down *in utero*) is virtually free of Pu compared with other calcified tissues that were formed after birth, when neonates start to incorporate Pu through inhalation.

Evaluation of the Pu distribution in human tissues requires a better understanding of the binding properties of Pu(IV) with the iron transport protein transferrin (TRF), because it is known that Pu^4+^ interacts with TRF in a similar way to Fe^3+^ (Ansoborlo et al. 2006; Bair and Thomson 1974; Duffield 2001; Krahenbuhl et al. 2005; Mason et al. 1992; Mecklenburg et al. 1997; Russell et al. 2003; Sikov and Kelman 1989; Taylor 1998). Recent discoveries about the metabolism of Fe in humans demonstrated the role of different proteins and divalent metal transporters in biologic membrane crossing (Donovan et al. 2000; Grossmann et al. 1998; Kaplan and Kushner 2000). All these studies conclude that Fe^3+^ binding to TRF induces overall conformational changes in the protein structure. These changes are required for TRF-bound iron to be recognized by surface cell receptors before Fe release. Although Pu binds strongly to TRF, it appears that it is unable to induce the required conformational changes. Accordingly, a stable Pu(IV)–TRF complex is observed until degradation of TRF releases Pu on the bone surface and in the liver (Durbin et al. 1997; Talbot et al. 1993). In fact, our results indirectly confirm that Pu is not really an active “bone seeker,” that is, does not replace Ca^2+^ in the crystalline structure of bone hydroxyapatite, in contrast to divalent metal cations such as Mg^2+^, Zn^2+^, or Pb^2+^. Rather, bones are a passive receptacle for Pu, which possibly precipitates as polymeric Pu(OH)_4_ (Ansoborlo et al. 2006; Duffield 2001).

Our long-term study looking at the effects of radioactive fallout from nuclear bomb testing on the Swiss population shows that the Pu present in the environment, unlike ^90^Sr, does not cross the placental barrier during pregnancy. Thus, Pu may not represent a radiologic risk for the radiosensitive tissues of a fetus. However, we did observe that children accumulate Pu in the bonelike root zone of the teeth (pulp and cementum) at the time they shed their primary teeth, with a comparable risk of accumulation in the bones. Similarly, we found that traces of fallout Pu accumulate in the vertebrae of adults.

## Figures and Tables

**Table 1:** SF-ICP-MS determination of Pu in bulk milk teeth.

Year of birth	^239^Pu (μBq/g Ca)	^240^Pu/^239^Pu atom ratio	Year of birth	^239^Pu (μBq/g Ca)	^240^Pu/^239^Pu atom ratio
1950–1951	4.7 ± 0.69	0.320 ± 0.045	1964	2.6 ± 0.34	^240^Pu < LD
1951	5.0 ± 0.59	0.202 ± 0.024	1965	1.7 ± 0.83	0.307^a^
1952	4.2 ± 0.61	0.186 ± 0.026	1965	1.6 ± 0.47	^240^Pu < LD
1953	6.9 ± 0.55	0.173 ± 0.009	1966	1.6 ± 0.6	^240^Pu < LD
1953	5.3 ± 0.68	0.187 ± 0.025	1966	3.1 ± 0.45	^240^Pu < LD
1954	6.5 ± 0.53	0.183 ± 0.010	1967	1.2 ± 0.29	^240^Pu < LD
1954	7.7 ± 0.84	0.167 ± 0.018	1968	1.1^a^	^240^Pu < LD
1955	7.7 ± 0.51	0.133 ± 0.007	1968	1.6 ± 0.5	^240^Pu < LD
1956	7.3 ± 0.79	0.234 ± 0.027	1969	2.2 ± 0.5	0.150 ± 0.008
1957	5.6 ± 1.27	0.227 ± 0.061	1969	3.4 ± 0.28	0.168 ± 0.050
1958	4.7 ± 0.79	0.263 ± 0.051	1970	3.9 ± 0.74	^240^Pu < LD
1959	4.7 ± 1.57	0.353^a^	1984–1985	1.4 ± 0.57	0.277 ± 0.26
1960	4.8 ± 1.18	0.333 ± 0.099	1986	0.5 ± 0.9	^240^Pu < LD
1960–1961	4.9 ± 1.08	0.214 ± 0.057	1987–1988	0.4^a^	^240^Pu < LD
1961	4.1 ± 0.54	0.167 ± 0.022	1995	0.6 ± 0.35	^240^Pu < LD
1962	3.1 ± 0.46	^240^Pu < LD	1953–1954^b^	49.8 ± 2.0	0.173 ± 0.017
1962	3.9 ± 1.08	0.103 ± 0.041	1953–1954^c^	5.9 ± 1.0	0.346 ± 0.138
1963	3.2 ± 0.40	^240^Pu < LD	1963–1965^b^	19.0 ± 1.3	0.182 ± 0.029
1964	4.2 ± 1.01	0.222 ± 0.064	1963–1965^c^	4.4 ± 0.6	0.232 ± 0.081

atom, atomic. For each measurement result, we estimated the combined standard uncertainty (± SD) by combining all the uncertainty components according to the law of propagation of uncertainty.

a SD exceeds the mean value by a factor of > 2.

b Root.

c Enamel.
